# Theoretical Investigation of Rate Coefficients and Dynamical Mechanisms for N + N + N Three-Body Recombination Based on Full-Dimensional Potential Energy Surfaces

**DOI:** 10.3390/molecules29204933

**Published:** 2024-10-18

**Authors:** Chong Xu, Zhenxuan Wei, Huayu Hu, Xixi Hu, Daiqian Xie

**Affiliations:** 1 Kuang Yaming Honors School, Nanjing University, Nanjing 210023, China; cxu23@smail.nju.edu.cn; 2 Institute of Theoretical and Computational Chemistry, Key Laboratory of Mesoscopic Chemistry, School of Chemistry and Chemical Engineering, Nanjing University, Nanjing 210023, China; 652023240012@smail.nju.edu.cn (Z.W.); dqxie@nju.edu.cn (D.X.); 3 Hypervelocity Aerodynamics Institute, China Aerodynamics Research and Development Center, Mianyang 621000, China; 4 National Key Laboratory of Aerospace Physics in Fluids, Mianyang 621000, China; 5 Hefei National Laboratory, Hefei 230088, China

**Keywords:** recombination mechanism, potential energy surface, three-body collision, excited states, rate coefficient, quasi-classical trajectory

## Abstract

Three-body recombination reactions, in which two particles form a bound state while a third one bounces off after the collision, play significant roles in many fields, such as cold and ultracold chemistry, astrochemistry, atmospheric physics, and plasma physics. In this work, the dynamics of the recombination reaction for the N_3_ system over a wide temperature range (5000–20,000 K) are investigated in detail using the quasi-classical trajectory (QCT) method based on recently developed full-dimensional potential energy surfaces. The recombination products are N_2_(*X*) + N(^4^*S*) in the 1^4^*A*″ state, N_2_(*A*) + N(^4^*S*) in the 2^4^*A*″ state, and N_2_(*X*) + N(^2^*D*) in both the 1^2^*A*″ and 2^2^*A*″ states. A three-body collision recombination model involving two sets of relative translational energies and collision parameters and a time-delay parameter is adopted in the QCT calculations. The recombination process occurs after forming an intermediate with a certain lifetime, which has a great influence on the recombination probability. Recombination processes occurring through a one-step three-body collision mechanism and two distinct two-step binary collision mechanisms are found in each state. And the two-step exchange mechanism is more dominant than the two-step transfer mechanism at higher temperatures. N_2_(*X*) formed in all three related states is always the major recombination product in the temperature range from 5000 K to 20,000 K, with the relative abundance of N_2_(*A*) increasing as temperature decreases. After hyperthermal collisions, the formed N_2_(*X*/*A*) molecules are distributed in highly excited rotational and vibrational states, with internal energies mainly distributed near the dissociation threshold. Additionally, the rate coefficients for this three-body recombination reaction in each state are determined and exhibit a negative correlation with temperature. The dynamic insights presented in this work might be very useful to further simulate non-equilibrium dynamic processes in plasma physics involving N_3_ systems.

## 1. Introduction

In the plasma flow of hypersonic spacecraft, the temperature can soar into tens of thousands of kelvins, and the strong temperature gradient often leads to thermochemical non-equilibrium phenomena. Under such extreme conditions, drastic collisions among particles, such as N_2_, O_2_, NO, N, and O, including chemical reactions, internal energy exchange, and recombination processes, take place all the time. These interactions are critical for evaluating a spacecraft’s thermal protection system and ensuring stable flight. Both experimental and theoretical characterizations of the dynamics of air plasmas over this vast temperature range are quite challenging due to the intricate interplay of various kinetics. Theoretically, computational fluid dynamics (CFDs) [1,2,3,4,5,6], direct Monte Carlo simulation [7,8], and direct molecular simulation [9,10,11,12] can be employed to model hypersonic flows, with the accuracy of these simulations relying heavily on the precise rate used to model hypersonic flow.

Due to its large abundance in the air, collision-induced reactions involving nitrogen species are crucial in many high-temperature systems. The availability of accurate ab initio potential energy surfaces (PESs) as well as appropriate reaction models for high-temperature dynamics, such as quasi-classical trajectory (QCT) methods, facilitates detailed investigation into such reactions. Recent research studies have focused on the high-temperature dynamics of the dissociation and exchange reactions involving ground-state nitrogen molecules, such as N_2_($X^{\;1}\Sigma_{g}^{+}$) + N(^4^*S*/^2^*D*) [13,14,15,16] and N_2_($X^{\;1}\Sigma_{g}^{+}$) + N_2_($X^{\;1}\Sigma_{g}^{+}$) [17], by performing QCT calculations based on ab initio PESs. However, recombination reactions have received comparatively less attention. Recently, Kondur et al. [18] investigated the recombination reaction dynamics of three N atoms to form N_2_($X^{\;1}\Sigma_{g}^{+}$) using the QCT method, yielding recombination rate coefficients on the order of 10^−45^ m^6^s^−1^ between 1000 and 18,000 K with a negative temperature dependence. It is noteworthy that the N_3_(^4^*A*″) PES they used was extracted from a ground singlet N_4_ PES developed at the CASPT2/AVTZ level of theory by placing one N atom far away from the others. However, Truhlar et al. [19] recently highlighted the inadequacy of this procedure to obtain N_3_(^4^*A*″) PESs, pointing out that the subsystem spin state cannot be controlled in a quartet for all configurations within the adiabatic ground-state calculations of the N_4_ system. Moreover, Geistfeld et al. [20] performed QCT calculations based on a new ground-state N_3_(^4^*A*″) PES constructed by Truhlar et al. [19], aiming to simulate the three-body collision of N atoms. They obtained recombination rate constants below 10^−45^ m^6^s^−1^ in the temperature range of 1000 to 10,000 K. 

In real gas environments, the three-body collision of three N(^4^*S*) atoms can occur in twelve spin states, including one detect, two octets, three sextets, four quartets, and two doublets. The ground quartet state for N_3_ considered in previous theoretical works only governs 1/16 of all N + N + N collisions. The recombination rate constants for other electronic states are still unavailable, primarily due to the absence of corresponding PESs. Furthermore, prior research has not concentrated on the formation of electronically excited N_2_ molecules, which are known to be prevalent and play a significant role in the energy transfer and complex formation processes within hypersonic flows. 

Recently, six global adiabatic PESs (four ^4^*A*″ states and two ^2^*A*″ states) of the N_3_ system were developed at the MRCI + Q/AVQZ level of theory [21]. These PESs are all correlated with the same N + N + N channel, revealing that atomic recombination is one of the formation pathways for the excited states of N_2_. Nonetheless, the kinetics and microscopic mechanisms underlying the formation of electronically excited nitrogen molecules during the three-body recombination of N_3_ systems remain elusive. Based on accurate PESs for relevant electronically excited states, the above information can be obtained by using an appropriate and accurate recombination reaction model to help us obtain a better understanding of the three-body collision dynamics for N_3_ systems. 

The QCT method is well suited for simulating the high-energy collision of N_3_ systems because of the relatively minor impact of quantum effects. Although the simulation of three-body collisions is inherently more complex than that of binary collisions, the QCT framework for examining recombination processes has been a subject of interest for many years [22,23,24,25,26,27]. Azriel et al. conceptualized the collision among three particles as a superposition of two binary collision processes, leading to the development of a QCT framework that was subsequently applied to investigate the collision of Cs^+^ and Br^−^ in a noble gas medium [25,26,27]. In their reaction model, a time-lag parameter that described the “approaching time” of the third body was introduced. A similar approach was adopted by Kondur et al. to explore the recombination dynamics in O_3_ [28], N_3_, and N_2_O [18] systems in only one electronic state.

Drawing on these studies, in this work, a time-delay parameter was also used in the recombination model, and the three-body recombination process of N + N + N → N_2_(*X*/*A*) + N was studied. The influences of different initial conditions on the distribution of the complex molecules were analyzed. The branching ratios and the rate coefficients forming different products from the three-body recombination reactions were discussed in detail. 

## 2. Computational Details

**Figure 1:**
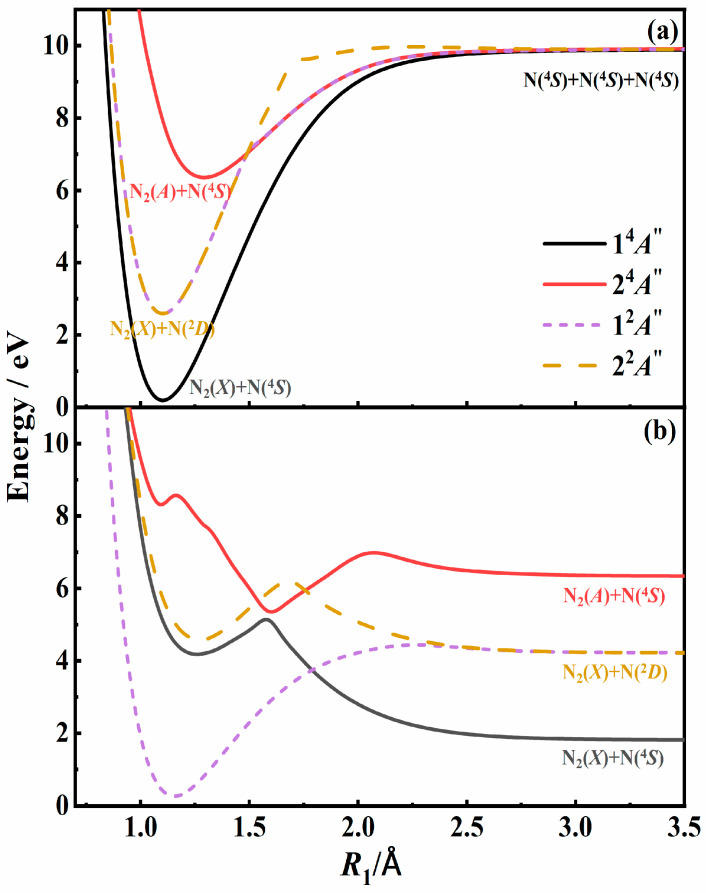
Potential energy curves as a function of *R*_1_ in the N_3_ system at the bond angle of 180°. (**a**) The third N atom is far away. (**b**) The other N-N distance *R*_2_ is equal to 1.293Å which is the equilibrium bond length of N_2_(*A*). The quartet and doublet states are shown in solid and dashed lines, respectively.

The QCT method was used to study the recombination pathways for N_3_ systems based on our recently developed four full-dimensional PESs covering both N + N_2_ and N + N + N dissociation channels [21]. Figure 1 shows the potential energy curves for two quartet and two doublet states for N_3_ systems as a function of N-N bond length (*R*_1_) with the N_a_-N_b_-N_c_ bond angle fixed at 180°. The third N atom is far away from the other two atoms in Figure 1a, and the other N-N distance (*R*_2_) is equal to the equilibrium bond length of the N_2_(*A*) molecule of 1.293Å in Figure 1b. The correlation diagram for the four states can be seen in the figure. As we can see, all of them are correlated to the N(^4^*S*) + N(^4^*S*) + N(^4^*S*) asymptotic region adiabatically. The ground 1^2^*A*″ state and excited 2^2^*A*″ state are correlated to the N_2_($X^{\;1}\Sigma_{g}^{+}$) + N(^2^*D*) asymptote, while the 1^4^*A*″ and 2^4^*A*″ states were correlated to the N_2_($X^{\;1}\Sigma_{g}^{+}$) + N(^4^*S*) and N_2_($A^{\;3}\Sigma_{u}^{+}$) + N(^4^*S*) channels, respectively. Among the adiabatic PESs, N_2_($A^{\;3}\Sigma_{u}^{+}$) can only be generated in the 2^4^*A*″ state, while N_2_(*X*) can be formed in the other three states. In addition, there are also many other states associated with N(^4^*S*) + N(^4^*S*) + N(^4^*S*) reactants. When calculating the branching ratios and rate coefficients, the electronic statistical weights of 2/64 for each doublet state and 4/64 for the quartet state should be considered according to the degeneracy of the electronic states. It was noteworthy that a conical intersection seam existed between the two quartet states, as depicted in Figure 1b, which suggested that it might be possible for the transition between the two states when three N atoms approached each other. However, nonadiabatic coupling calculations, which are challenging for the N_3_ system, have not been included and will be addressed in future investigations focusing on nonadiabatic dynamics.

For N_3_ systems, the recombination reaction in the three-body collision is shown as

N^A^ + N^B^ + N^C^ ⇋ N_2_ + N^i^, (i = A, B, C) (1)

where N^i^ (i = A, B, C) can carry excess energy, thus allowing the formation of the bound state N_2_. The recombination event can occur through one step when all three particles enter the interaction region simultaneously with low probability. The recombination process can also proceed via a two-step binary collision, in which the first collision producing an intermediate with a finite lifetime can be represented as follows:

N^A^ + N^B^ ⇋ ^*^N^A^N^B^ (2)

where the species marked with * were the intermediate. The intermediate collides with the third atom producing the complex. Depending on the role of the third atom or the components of the complex molecule, the two-step binary collision can be classified into two mechanisms. The two-step transfer mechanism, in which the third atom simply carries the excess energy from the intermediate to form a stable molecule, is expressed as follows:

^*^N^A^N^B^ + N^C^ ⇋ N^A^N^B^ + N^C^ (3)

In the other case shown below, the third atom actively replaces an atom in the intermediate to form a new molecule,

^*^N^A^N^B^ + N^C^ ⇋ N^A^N^C^ + N^B^ (4)

known as the two-step exchange mechanism. 

Given that the one-step three-body direct recombination process can be regarded as an extreme case of the two-step binary collision with a very small time delay, only the two distinct binary collision mechanisms were discussed in detail.

### 2.1. Binary Collision Parameters

Since a three-body collision process can be regarded as two binary collision processes, it requires information about the intermediate. The intermediate N_2_, resulting from the interaction between two N atoms, possessed a certain lifetime to interact with the third N atom. The scattering angle provided quantitative indicators of the overall deflection of the relative velocity vector in binary collisions, serving as a critical parameter for identifying collisions that lead to recombination. The scattering angle *χ* of the binary collision in a spherically symmetric potential *U*(*r*) is defined by the following equation:

(5) $$\chi (b,\varepsilon_{tr})=\pi -2b\int_{R_{\min}}^{\infty }\frac{dr/r^{2}}{\sqrt{1-\frac{b^{2}}{r^{2}}-\frac{U(r)}{\varepsilon_{tr}}}}$$

where *r*, *R*_min_, *b,* and *ε_tr_* represent the distance between the two N atoms, the closet distance, the impact parameter, and the relative translational energy, respectively. To quantify *U*(*r*), the N_2_ interaction potential was extracted from our N_3_ PESs by placing one N atom away from the rest, as shown in Figure 1a. Two N atoms were initially placed at a distance of 10 Å with an initial relative velocity determined by *ε_tr_*. The third atom was placed 100 Å away from the center of mass (COM) of the first two atoms, which did not promote the binary interaction. The impact parameter *b* was sampled uniformly between 0 and *b*_max_. When the distance was more than 30 Å, the simulation was stopped.

**Figure 2:**
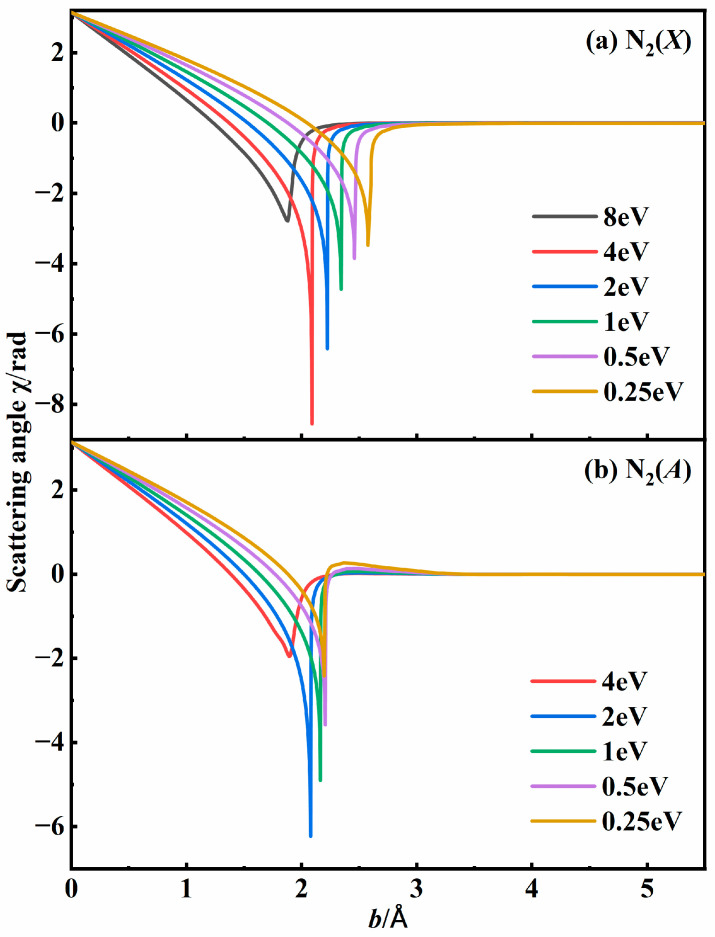
Scattering angle profiles in (**a**) N_2_(*X*) state and (**b**) N_2_(*A*) state for N + N system at specific relative translational energies based on N_3_ PESs.

Figure 2 shows the scattering angle for the N + N system at various relative translational energies based on PESs for both the N_2_(*X*) state and the N_2_(*A*) state. For the fixed energies with low impact parameters, the scattering angle *χ* was close to π, indicating a process when two atoms collide and then bounce off each other. When the impact parameter is larger than 3 Å, the interaction can be negligible, and *χ* approaches 0. When two atoms collide with a moderate impact parameter, the scattering angle is influenced by both the collisional energy and the PESs. It was worth noting that the scattering angle decreased sharply to form a singularity at some suitable impact parameter for a specific collisional energy. This singularity, characterized by a large negative scattering angle, is indicative of intermediate formation. Under the same initial conditions, the maximum scattering angle for the N_2_(*X*) state was greater than that for the N_2_(*A*) state. This is because the potential well of the *X* state was much deeper than that of the *A* state, as depicted in Figure 1a. The two N atoms rotated around their COM with a finite lifetime, and will eventually dissociate. Although the internal energy of the N_2_ molecule exceeds its dissociation energy, the diatomic molecule can still be trapped due to the existence of a centrifugal barrier.

The lifetime of the intermediate plays a crucial role in the recombination process and can be computed as follows:

(6) $$\tau (\varepsilon_{tr},b)=2\int_{R_{\min}}^{R_{\max}}{\left(\frac{\mu }{2}\right)}^{\frac{1}{2}}{\left[\varepsilon_{tr}(1-\frac{b^{2}}{r^{2}})-U(r)\right]}^{-\frac{1}{2}}dr$$

where *R*_min_ was the closest distance between the two atoms, *R*_max_ was the distance where the radial force between the two atoms was zero, and *μ* was the reduced mass of the binary system. 

**Figure 3:**
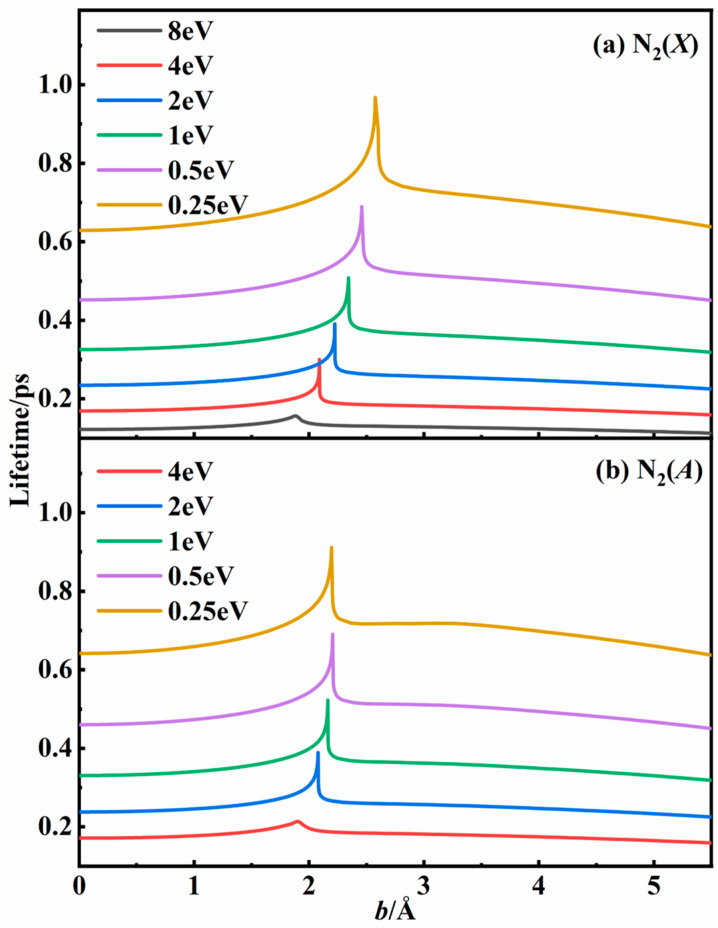
The lifetimes of intermediates as a function of impact parameter in (**a**) the N_2_(*X*) state and (**b**) the N_2_(*A*) state for the N + N system at specific relative translational energies based on the N_3_ PESs.

Figure 3 shows the lifetime of the N_2_ intermediate as a function of the impact parameter at different energy values. The lifetimes of the intermediates produced by binary collision under specific conditions varied from 0.1 ps to 1 ps, and they notably decreased as the collisional energy increased. For specific energy, the lifetime reached the maximum value when the impact parameter was near the singularity shown in Figure 2, indicating the formation of an intermediate. It is more likely to collide with the third body when the lifetime is longer. By analyzing the above data, we can develop an appropriate classical trajectory model to simulate the three-body collision process with greater precision.

### 2.2. Three-Body Collision Parameters

**Figure 4:**
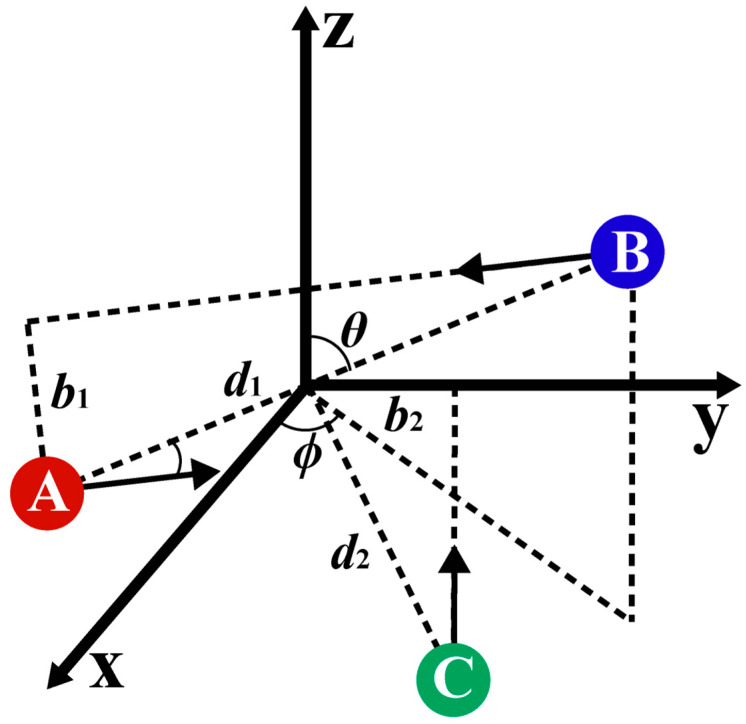
A schematic of a three-body interaction in QCT calculations. This image shows the kinematic parameters for the first collision between A and B atoms and the second collision between the COM of the pair AB and the C atom.

The pivotal dynamics parameters in a three-body collision are the impact parameters and relative translational energy, both of which are contingent upon the initial positions and velocities of atoms, as well as the PESs of the system. Following the previous work, the collisions between three particles were considered as two-step binary collisions, as depicted in Figure 4, where the first collision occurred between A and B atoms, and the second collision occurred between the C atom and the COM of AB. For the first binary collision system, the COM of AB was placed at the origin of the cartesian coordinate. The angle between the AB line and the *z*-axis was *θ*. The angle between the projection of the AB line in the *xy* plane and the *x*-axis was *ϕ*. The distance between the two atoms was *d*_1_. Therefore, the position of the two atoms can be determined, as shown in Equations (7a) and (7b). In the second binary collision system, the distance from the C atom to the COM of AB was *d*_2_. Each binary collision process had its impact parameter and relative translational energy. Therefore, two sets of the impact parameters (*b*_1_, *b*_2_), the relative translational energies (*E*_1_, *E*_2_), and the initial distances (*d*_1_, *d*_2_) should be used to describe a three-body collision process. In addition, three additional angles *θ*, *ϕ*, and *η* are required to completely specify the coordinates and momenta of all three atoms in space, which can be sampled from a uniform distribution. Two sets of the impact parameters were sampled from the distribution of *b*_i_ = *b*_imax_$\sqrt{\beta }$, where *β* is a uniform random number ranging from 0 to 1.

In this model, *μ*_1_ is the reduced mass of A and B atoms, and *μ*_2_ is the reduced mass of the C atom and the COM of pair AB for the second collision. Because the spatial position of A and B atoms was in random distribution, the initial position of the C atom can be placed in some plane containing the origin. In order to further eliminate the repeated position sampling, the position of the C atom was put in the region of y > 0, z < 0, and x = 0, of which the initial movement direction was limited towards the positive direction of the z-axis, which is opposite to the direction of AB motion. Thus, the spatial coordinate of the three atoms can be determined as follows:

(7a) $$A:\left(\begin{array}{c} x_{1}\\ x_{2}\\ x_{3} \end{array}\right)=\left(\begin{array}{c} -\frac{1}{2}d_{1}\sin\theta \cos\phi \\ -\frac{1}{2}d_{1}\sin\theta \sin\phi \\ -\frac{1}{2}d_{1}\cos\theta \end{array}\right)$$

(7b) $$B:\left(\begin{array}{c} x_{4}\\ x_{5}\\ x_{6} \end{array}\right)=\left(\begin{array}{c} \frac{1}{2}d_{1}\sin\theta \cos\phi \\ \frac{1}{2}d_{1}\sin\theta \sin\phi \\ \frac{1}{2}d_{1}\cos\theta \end{array}\right)$$

(7c) $$C:\left(\begin{array}{c} x_{7}\\ x_{8}\\ x_{9} \end{array}\right)=\left(\begin{array}{c} 0\\ b_{2}\\ -{\left(d_{2}^{2}-b_{2}^{2}\right)}^{1/2} \end{array}\right)$$

Due to the aforementioned restrictions on the directions of the second collision, the motion directions of A and B atoms need to be sampled throughout space. Given the relative translational energy *E*_1_ of the first collision, A and B atoms, respectively, acquire initial momenta of magnitude (2*μ*_1_*E*_1_)^1/2^ in opposite directions to keep their COM at the origin. The impact parameter between A and B was *b*_1_, which determined the angle between the initial momentum direction and the AB line. To cover all the momentum directions in the spatial sampling, the rotation angle about the AB line was *η*. It was difficult to determine the mathematical representations of the direction of AB momentum in the *xyz* coordinate system because the direction of AB changes. Three Euler angles *ϕ*, *θ,* and *η* were used to transform the coordinate system. 

To further avoid redundant sampling, the range of the angle *θ* was limited to [0, π], *ϕ* to [0, π/2], and *η* to [0, 2π]. The relative translational energies *E*_1_ and *E*_2_ of the two collisions followed a *Maxwell–Boltzmann* distribution at a particular temperature. The parameters *d*_1_, *d*_2_, *θ*, *ϕ*, and *η* were sampled by the Monte Carlo method.

Once all the motion parameters were initialized, it was obvious that increasing *d*_2_ would delay the time for the C atom to interact with other atoms. Thus, the time delay is an additional degree of freedom that needs to be taken into account in the three-body collision process. The following time-delay parameter is used to quantify this delay effect:

(8) $$\omega =\frac{t_{2}-t_{1}}{\tau (E_{1})}$$

in which *t*_1_ and *t*_2_ were the lifetimes of the first and second collisions, respectively. The normalized lifetime *τ*(*E*_1_) was the average lifetime of the intermediate in the first collision under a specific kinetic energy. The value of *ω* of the one-step direct three-body collision was zero, while for the two-step binary collision, *ω* had a nonzero value. The positive value of *ω* means that the C atom lags behind the interaction of A and B, and the negative value can be explained by C interacting with A or B before the interaction between A and B. Thus, the theoretical framework treated one-step three-body direct recombination as an extreme case of two-step binary collisions and allowed for a unified treatment of all recombination mechanisms.

In the QCT calculations, three-body collision processes have been simulated in the 1^4^*A*″, 2^4^*A*″, 1^2^*A*″, and 2^2^*A*″ states at 5000 K, 10,000 K, 15,000 K, and 20,000 K, with 10 million trajectories at each temperature. The recombination probability of the three-body collision is highly sensitive to the input parameters. So, it is important to set up reasonable parameters to obtain accurate results. The initial distances of *d*_1_ and *d*_2_ of two binary collisions were distributed within the range of 10–20 Å. After extensive tests, the maximum impact parameters of the two collision processes *b*_1max_ and *b*_2max_ of each electronic state were set to the same value of 5.5 Å at different temperatures. The trajectory evolution stopped when the overall reaction time exceeded a time threshold of 100 ps or when the maximum distance between any two atoms exceeded 100 Å. Based on these trajectories, a thorough analysis of the three-body recombination mechanisms, impact parameter distributions, vibrational and rotational quantum number distributions, and rate coefficients was conducted. 

### 2.3. Rate Coefficients

Analogous to the two-body recombination rate coefficient, the three-body recombination rate coefficient in each state can be expressed as follows [28]:

(9) $$\begin{array}{l} k(T)=\frac{1}{2\tau }\int_{E_{1}=0}^{\infty }\int_{E_{2}=0}^{\infty }\int_{\omega_{\min}}^{\omega_{\max}}\int_{b_{1}=0}^{b_{1\max}}\int_{b_{2}=0}^{b_{2\max}}\frac{f(E_{1})}{\mu_{1}}\frac{f(E_{2})}{\mu_{2}}(2\pi b_{1})(2\pi b_{2})\\ |\omega |\tau (E_{2})f_{\omega }(\omega )P(E_{1},E_{2},b_{1},b_{2},\omega )db_{2}db_{1}d\omega dE_{2}dE_{1} \end{array}$$

where *ω*_min_ and *ω*_max_ were the minimum and maximum values of the time-delay parameters, and *f*(*E*_i_) was the distribution of the relative translational energy given by the *Maxwell–Boltzmann* distribution. The probability of the successful three-body recombination was calculated by the number of recombination trajectories divided by the total trajectory number. And *τ* was the average lag time in the three-body collision, which is difficult to determine from kinetic theory. In the studies of O_3_ and N_3_ systems, *τ* was set to a constant value of 10 ps. In this work, *τ* was also treated as the same constant for simplicity. *f_ω_*(*ω*) was the distribution of the time-delay parameter decided by the statistical results.

### 2.4. Binning Method

The internal energy for a molecule can be divided into vibrational energy and rotational energy. The rotational quantum number of produced molecules can be easily obtained from diatomic angular momentum using the following:

(10) $$|\vec{j}|=\sqrt{J(J+1)}\hbar$$

The Einstein–Brillouin–Keller (EBK) method [29] can be used to determine the vibrational quantum number of a diatomic product. The vibration quantum number can be expressed as integral semiclassical quantization:

(11) $$(v+\frac{1}{2})h=\oint \left\{2\mu [E-V(r)-J(J+1)\hbar^{2}/2\mu r^{2}\right]{\}}^{1/2}dr$$

where *μ* is the reduced mass and *h* is Planck’s constant. 

## 3. Results and Discussion

### 3.1. Recombination Mechanisms

**Figure 5:**
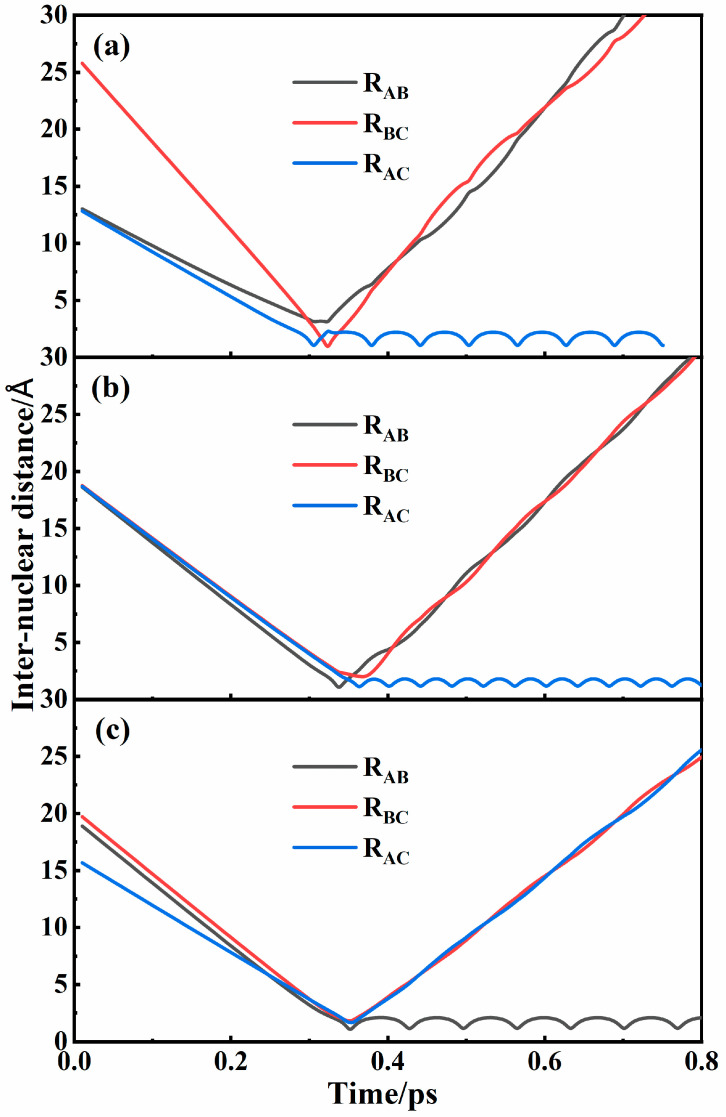
Typical recombination trajectories for (**a**) two-step transfer mechanism, (**b**) two-step exchange mechanism, and (**c**) one-step three-body direct recombination mechanism in 2^4^*A*″ state of N_3_ systems.

Numerous trajectories have been performed on the four PESs. Initially, the interatomic distances were set sufficiently large to preclude any interactions. The successful recombination of N_2_ can be characterized by the temporal periodicity of the internuclear distance which represents the stretching vibration of a diatomic molecule. Figure 5 shows three representative recombination trajectories corresponding to different mechanisms. As shown in Figure 5a, A and C atoms interacted to form an intermediate that has a certain lifetime. During the lifetime, the B atom interacted with the intermediate and took away some energy, leading to the formation of a new diatomic molecule AC. Figure 5b also shows the formation of the AC molecule, but the intermediate AB was formed first, which was different from the situation in Figure 5a. Because of the obvious time lag between the two kinds of interactions, both of them corresponded to the two-step binary collision mechanism. The intermediate and the newly formed molecule were the same in Figure 5a and were different in Figure 5b, which corresponded to the two-step transfer mechanism and the two-step exchange mechanism, respectively. In Figure 5c, it can be clearly seen that there was no significant time lag between the two collisions and that the formation of an intermediate occurred almost simultaneously with the formation of a new recombination molecule. It can be considered that the three atoms interact simultaneously to form a new molecule by a one-step three-body direct recombination mechanism. All recombination mechanisms can be found in the collisions in each state. Because of the low probability of three-body direct recombination, the mechanisms for the two-step binary collision process were distinguished and discussed in detail below. Only collisions in the 2^4^*A*″ state can form N_2_(*A*) molecules, while the three other states (1^4^*A*″, 1^2^*A*″, and 2^2^*A*″) yielded N_2_(*X*) molecules. 

**Figure 6:**
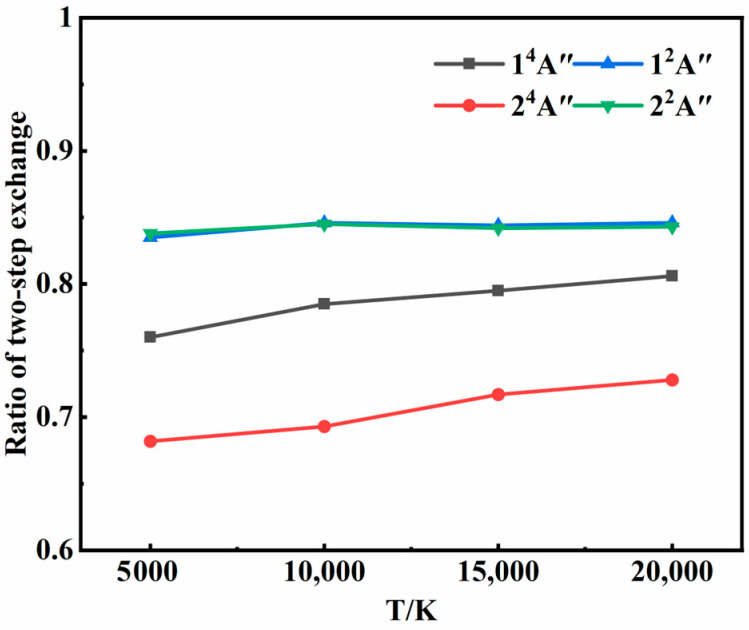
Ratios of two-step exchange mechanism at different temperatures in each state.

Figure 6 shows the ratios of the two-step exchange mechanism at different temperatures. For each state, the two-step exchange mechanism was overwhelmingly dominant and increased with temperature, suggesting higher energy conversion efficiency for atom exchange processes compared to inelastic collisions. With the increase in temperature, the initial translational energy of the atoms increased, shortening the average collision time. It became less probable for the third atom to remove excess energy through energy transfer. Instead, it was more likely to collide with an atom of the intermediate, facilitating exchange and the formation of a new molecule. This trend aligned with previous studies [30,31] utilizing isotope labeling, which found that exchange collisions between O_2_ and O typically resulted in more significant internal energy changes than inelastic collisions. In addition, it was found in the two-step transfer mechanism that the energy transfer process often preceded intermediate formation, corresponding to scenarios of *ω* < 0. In other words, the third atom transferred some energy from one of the atoms that were going to be involved in forming the intermediate, and then the intermediate was formed.

### 3.2. Recombination Probabilities

**Figure 7:**
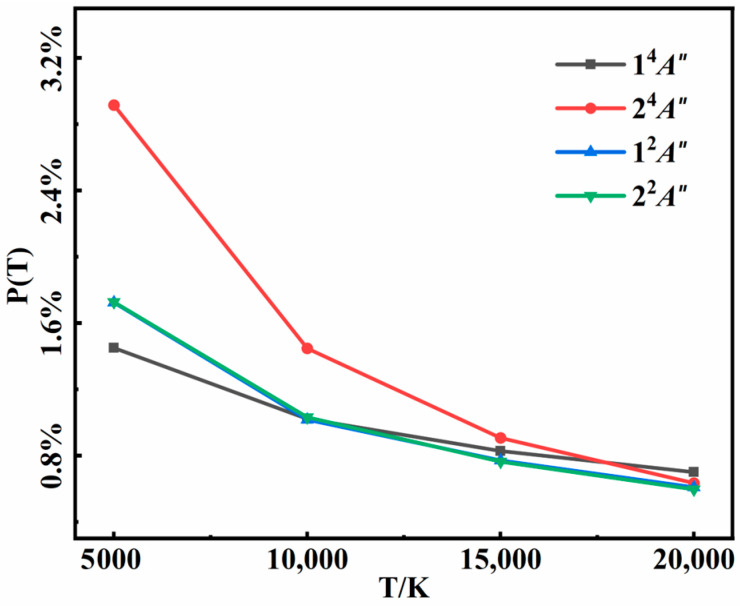
Recombination probabilities of the three-body collision for 1^4^*A*″, 2^4^*A*″, 1^2^*A*″, and 2^2^*A*″ states at different temperatures.

The recombination products included N_2_(*X*) + N(^4^*S*) in the 1^4^*A*″ state, N_2_(*A*) + N(^4^*S*) in the 2^4^*A*″ state, and N_2_(*X*) + N(^2^*D*) in both 1^2^*A*″ and 2^2^*A*″ states in our calculations. The probabilities of successful three-body recombination as a function of temperature in the four electronic states are shown in Figure 7. In general, the total probability of recombination decreased as the temperature increased. Higher collisional energy at higher temperatures not only hindered the formation of an intermediate but also diminished the likelihood of the second collision. On the other hand, higher collision energy often resulted in a shorter intermediate lifetime, narrowing the time window for the second collision and reducing the three-body recombination probability. The recombination probabilities in the 1^2^*A*″and 2^2^*A*″ states were essentially identical, as both the two states correlated to the same N_2_(*X*) + N(^2^*D*) asymptote. For the two quartet states, it was surprising that the probability in the 2^4^*A*″ state was twice as much as it was in the 1^4^*A*″ state, even though the formed diatomic molecule N_2_(*X*) had much larger dissociation energy than N_2_(*A*). In addition, recombination probability in the 2^4^*A*″ state exhibited a more pronounced temperature dependence than the others. Accounting for state degeneracy, N_2_(*X*) was always the major recombination product in the temperature range of 5000 K to 20,000 K, but the branching ratio of N_2_(*A*) became larger and larger as the temperature decreased.

**Figure 8:**
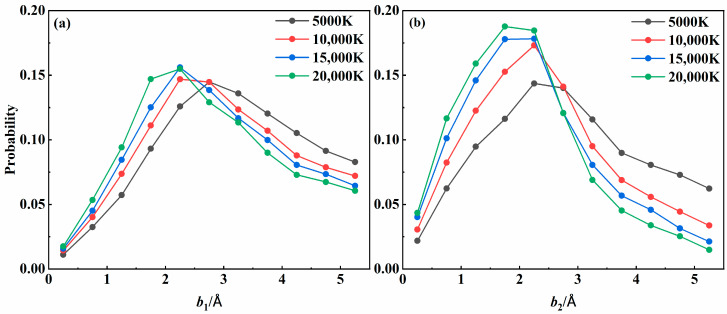
Recombination probability distribution with impact parameters (**a**) *b*_1_ and (**b**) *b*_2_ for the 2^4^*A*″ state at various temperatures.

To deeply understand the recombination dynamics for three-body collisions, the recombination probabilities were plotted as a function of impact parameters *b*_1_ and *b*_2_ for the 2^4^*A*″ state at four different temperatures in Figure 8. Overall, the recombination probability increased with *b*_1_ initially and then decreased. It was found that collisions with an impact parameter corresponding to the longer lifetime value shown in Figure 3 were more likely to result in recombination. As the temperature increased, the *b*_1_ value that yields the largest recombination probability gradually decreased, consistent with the temperature dependence of *b*_1_ in forming an intermediate with the longest lifetime. In addition, the distribution of *b*_2_ was more concentrated in the lower range than that of *b*_1_. Since collisions with lower impact parameters can transfer energy more efficiently, they were expected to dominate the recombination process, becoming more critical as the temperature increased.

**Figure 9:**
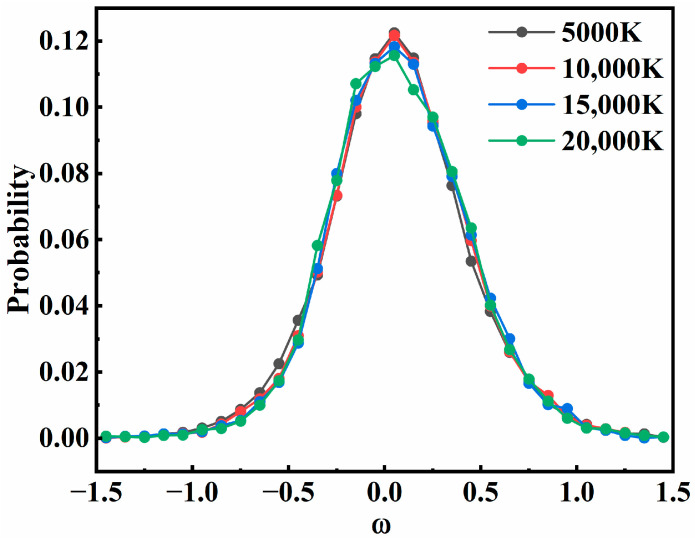
The distribution of *ω* for successful recombination of the 2^4^*A*” state at different temperatures.

In this model, the influence of different values of *d*_1_ and *d*_2_ was normalized to the time-delay parameter *ω*. Then, the effect of the time-delay parameter on overall recombination probability was examined, as shown in Figure 9, where the distribution of *ω* at four different temperatures was plotted. All curves in the figure show that the recombination probability had a strong dependence on *ω*. As a whole, there was a correlation between the two collisions. The closer the two collisions occurred, the more likely it was for recombination to occur. Most of the successful recombination trajectories were distributed in the *ω*∈[−0.5, 0.5]. As the value of |*ω*| increased, the recombination probability decreased sharply, because the large time-delay parameter left only a few chances for the third atom to collide. This suggested that collisions with very low time-delay parameters can more effectively realize the recombination results. The effect of the sequence of the two collisions on the recombination probability is not strictly symmetric, with a slight preference for intermediate formation before the second collision, which was also consistent with the proportion of the two-step recombination mechanisms shown in Figure 6. The same feature was also observed in other states.

### 3.3. Quantum State Distribution

**Figure 10:**
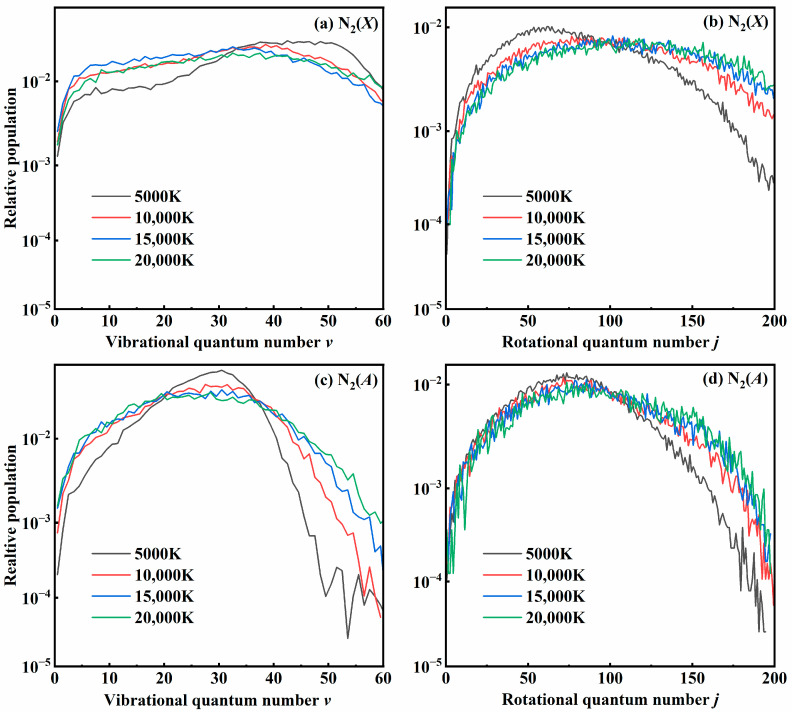
The distributions of (**a**,**c**) vibrational states and (**b**,**d**) rotational states of N_2_(*X*) and N_2_(*A*) products at different temperatures.

The formation of diatomic products is accompanied by a significant release of energy, leading to a high level of internal excitation. Distributions of the vibrational and rotational quantum numbers of both N_2_(*X*) and N_2_(*A*) states at different temperatures are shown in Figure 10. It is important to point out that our results for N_2_(*X*) for the 1^4^*A*″ state align well with those previously reported by Kondur et al. [18]. The distributions for N_2_(*X*) shown in Figure 10a,b represent a cumulative result for the three relative states. Obviously, at high temperatures, N_2_(*X*) molecules have been highly excited in both rotational and vibrational states; however, as the temperature drops to 5000 K, the vibrational distributions become hotter and the rotational distribution is colder. For N_2_(*A*), the excitation is less intense than the results for N_2_(*X*) due to less exothermicity for the recombination reaction. As the temperature increases, both distributions become much flatter, leading to a larger population of high *ν* and low *ν*, along with a rise in the number of highly rotationally excited molecules. In the second high-energy binary collision, one atom of the intermediate is impacted by the third atom, leading to the formation of a recombination molecule with a high rotational excitation. This is consistent with the early reports of the dissociation behavior of the N_2_ molecule.

### 3.4. Rate Coefficients

**Figure 11:**
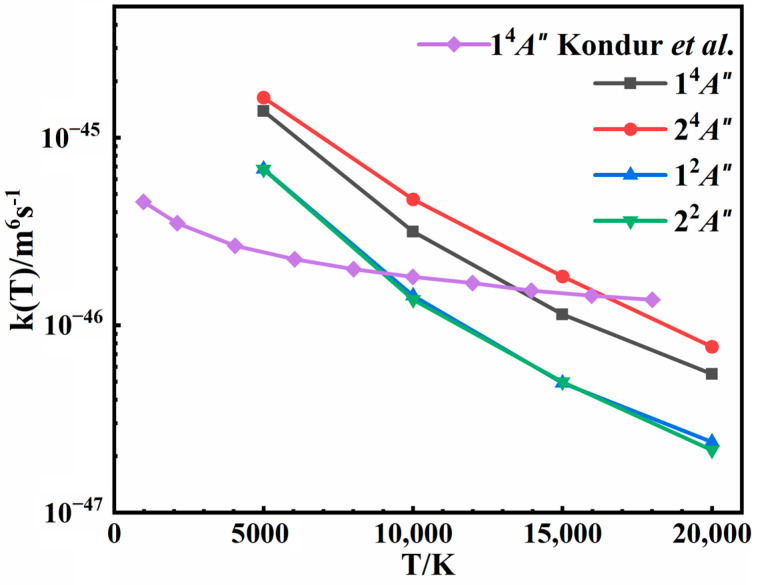
Rate coefficients of the three-body recombination for 1^4^*A*″, 2^4^*A*″, 1^2^*A*″, and 2^2^*A*″ states at different temperatures. The results of Kondur et al. [18], multiplied by a weight of 3/16, are also plotted for comparison.

The rate coefficients, taking into account the weights of 1/16 for the quartet state and 1/32 for the doublet state, are shown in Figure 11, with their absolute values detailed in Table 1. It is evident that the calculated recombination rate coefficients are negatively correlated with temperature, aligning with previous results [18,20]. An increase in temperature leads to higher collision energy, which reduces the lifetime of the intermediate. This reduction in the lifetime decreases the likelihood of three-body collisions and the probability of recombination, consequently lowering the rate coefficient. For the 1^4^*A*″ state, despite the use of similar QCTs in both studies, our results show a more pronounced temperature dependence and differ less than an order of magnitude from the recent theoretical results obtained from Kondur et al. [18], as shown in Figure 11. The PES they used was derived from a ground singlet N_4_ PES developed at the CASPT2/AVTZ level of theory by placing one N atom far away from the others, which does not always maintain a quartet spin state for all configurations. Thus, the differences in the rate coefficients are mainly due to the difference in the PESs. Furthermore, the results for the other three states are reported in this work. It is worth noting that the 2^4^*A*″ state which forms the N_2_(*A*) product has larger recombination rate coefficients than the 1^4^*A*″ state producing N_2_(*X*). This finding suggests a potentially significant role for N_2_(*A*) in hypersonic flows. As far as we know, the analytical temperature dependences of the rate coefficients for these processes were unknown before, particularly for the excited states. Consequently, the kinetic information reported in this work is expected to be instrumental in enhancing the simulation models for high-temperature aerodynamics.

## 4. Conclusions

In this study, the recombination dynamics of the three-body collision of the N_3_ system from 5000 to 20,000 K were calculated by using a three-body collision model in the QCT framework based on our recently developed four PESs. Our results indicate that the recombination process is primarily governed by the intermediate species produced during the initial binary collision and is substantially affected by the time-delay parameters. The recombination products are N_2_(*X*) + N(^4^*S*) in the 1^4^*A*″ state, N_2_(*A*) + N(^4^*S*) in the 2^4^*A*″ state, and N_2_(*X*) + N(^2^*D*) in both the 1^2^*A*″ and 2^2^*A*″ states. Throughout the examined temperature range, a two-step exchange mechanism was observed to be dominant in all states. The calculated recombination rate coefficients for three-body collisions in each state exhibit a negative correlation with temperature. Furthermore, the recombination products N_2_(*A*) and N_2_(*X*) have comparable branching ratios, and show a strong non-Boltzmann behavior in quantum state populations with high vibrational excitation and relatively lower rotational excitation. 

This study provides a comprehensive understanding of the three-body collision of the N_3_ system in several electronic states. It elucidates the underlying reaction mechanisms and identifies the pivotal factors that shape the collision outcomes. Furthermore, the quantum state distributions of the products, along with the state-specific rate coefficients, provide valuable kinetic data for the simulation of plasma flows involving nitrogen species at high temperatures. It is important to acknowledge that nonadiabatic couplings between these electronic states, which present a considerable computational challenge, were not considered in our current calculations. These couplings have the potential to significantly shift the conclusions derived from this study, so they warrant thorough investigation in forthcoming research works.

## Figures and Tables

**Table 1:** Rate coefficients (unit in m^6^s^−1^) of the three-body recombination for 1^4^*A*″, 2^4^*A*″, 1^2^*A*″, and 2^2^*A*″ states at different temperatures.

	T/K.	5000	10,000	15,000	20,000
States					
**1^4^*A*″**		1.3876 × 10^−45^	3.1876 × 10^−46^	1.1423 × 10^−46^	5.4983 × 10^−47^
**2^4^*A*″**		1.6365 × 10^−45^	4.6891 × 10^−46^	1.8215 × 10^−46^	7.6794 × 10^−47^
**1^2^*A*″**		6.8119 × 10^−46^	1.4345 × 10^−46^	4.9191 × 10^−47^	2.3819 × 10^−47^
**2^2^*A*″**		6.7822 × 10^−46^	1.3682 × 10^−46^	4.9828 × 10^−47^	2.1555 × 10^−47^
**Total**		4.3835 × 10^−45^	1.0650 × 10^−45^	3.9540 × 10^−46^	1.7716 × 10^−46^

## Data Availability

The data that support the findings of this study are available upon reasonable request from the corresponding author X. Hu (xxhu@nju.edu.cn).
